# Global perspectives on MASCC clinical practice guidelines: awareness, implementation and insights to inform future updates

**DOI:** 10.1007/s00520-026-11388-1

**Published:** 2026-11-04

**Authors:** Ragda Abdalla-Aslan, Paola Alberti, Nicole Blijlevens, Pierluigi Bonomo, Joanne Bowen, Julia S. Bruno, Raymond J. Chan, Yin Ting Cheung, Andrew Davies, Mellar Davis, Sharon Elad, Joel Epstein, Darren Fernandes, Jason L. Freedman, Nicolas H. Hart, Hirotoshi Iihara, Michael Jefford, Daniel Peter Johnstone, Amber S. Kleckner, Bogda Koczwara, Grigorios Kotronoulas, Emma Munro, Barbara Murphy, Midori Nakagaki, Enrique Soto-Perez-de-Celis, Stephen Sonis, Nikolaos Tsoukalas, Anna Ugalde, Corina van den Hurk, Hannah Rose Wardill, Paolo Bossi

**Affiliations:** 1 Technion Israel Institute of Technology, Faculty of Medicine, Technion City, Haifa, 3200003 Israel; 2 Department of Oral and Maxillofacial Surgery, Rambam Health Care Campus, Haifa, Israel; 3 School of Medicine and Surgery, University of Milano-Bicocca, Monza, Italy; 4 Department of Hematology, Radboud University Medical Centre, Nijmegen, The Netherlands; 5 Radiation Oncology, Azienda Ospedaliero-Universitaria Careggi, Florence, Italy; 6 School of Pharmacy and Biomedical Science, Adelaide University, Adelaide, Australia; 7 Instituto de Ensino E Pesquisa, Hospital Sírio-Libanês, São Paulo, Brazil; 8 Flinders LiveWell Cancer Research Centre, Caring Futures Institute, College of Health and Enablement, Flinders University, Adelaide, SA Australia; 9 School of Pharmacy, Faculty of Medicine, the Chinese University of Hong Kong, Shatin, Hong Kong SAR Hong Kong; 10 School of Medicine, Trinity College Dublin, Dublin, Ireland; 11 Palliative Medicine, Division of Oncology Supportive Care, Levine Cancer Institute, Charlotte, NC USA; 12 Oral Medicine, Eastman Institute for Oral Health, University of Rochester Medical Center, Rochester, NY USA; 13 Cedars Sinai National Medical Center Los Angeles, Los Angeles, CA USA; 14 Cancer Dentistry, Division of Otolaryngology and Head and Neck Surgery, City of Hope, Duarte, CA USA; 15 The Department of Gastroenterology, Nottingham Digestive Diseases Centre, Derby Road, Nottingham, NG7 2UH UK; 16 Division of Oncology, Children’s Hospital of Philadelphia, Department of Pediatrics, Perelman School of Medicine, University of Pennsylvania, Philadelphia, PA USA; 17 Human Performance Research Centre, INSIGHT Research Institute, University of Technology Sydney (UTS), Sydney, NSW Australia; 18 Department of Pharmacy, Gifu University Hospital, 1-1 Yanagido, Gifu, 501-1194 Japan; 19 Cancer Center, Gifu University Hospital, 1-1 Yanagido, Gifu, 501-1194 Japan; 20 Centre for Health Services Research in Cancer, Australian Cancer Survivorship Centre, and Department of Medical Oncology, Peter MacCallum Cancer Centre, Melbourne, Victoria Australia; 21 Sir Peter MacCallum Department of Oncology, Peter MacCallum Cancer Centre, Melbourne, Victoria Australia; 22 Adelaide University, Adelaide, Australia; 23 Department of Pain and Translational Symptom Science, University of Maryland School of Nursing, Baltimore, MD 21201 USA; 24 Australian Research Centre for Cancer Survivorship, UNSW Medicine & Health, UNSW Sydney, in partnership with Cancer Council NSW, Sydney, Australia; 25 Flinders University, Adelaide, Australia; 26 School of Medicine, Dentistry and Nursing, University of Glasgow, Glasgow, UK; 27 Haematology and Bone Marrow Transplant Cancer Care Services, Royal Brisbane and Women’s Hospital, Herston, Queensland Australia; 28 Vanderbilt University Medical Center, Nashville, TN USA; 29 Pharmacy Department, Royal Brisbane and Women’s Hospital, Herston, Queensland Australia; 30 Division of Medical Oncology, University of Colorado Anschutz Medical Campus, Aurora, CO USA; 31 Divisions of Oral Medicine, Brigham and Women’s Hospital and Dana-Farber Cancer Institute, Boston, MA USA; 32 Biomodels. LLC and Primary Endpoint Solutions, LLC, Waltham, MA USA; 33 Oncology Department, Veterans Hospital (417 NIMTS), Athens, Greece; 34 Henry Dunant Hospital Center, Athens, Greece; 35 Institute for Health Transformation, Quality and Patient Safety, Deakin University, Geelong, Victoria Australia; 36 Catharina Hospital, Eindhoven, The Netherlands; 37 Santeon, Utrecht, The Netherlands; 38 School of Pharmacy and Biomedical Science, College of Health, Adelaide University, Adelaide, South Australia Australia; 39 Supportive Oncology Research Group, Precision Cancer Medicine Theme, South Australian Health and Medical Research Institute, Adelaide, South Australia Australia; 40 Department of Biomedical Sciences, Humanitas University, IRCCS Humanitas Research Hospital, Milan, Italy

**Keywords:** Practice guideline, Guideline adherence, Supportive care, Evidence-based practice, Clinical protocols, Cancer

## Abstract

**Purpose:**

The Multinational Association of Supportive Care in Cancer (MASCC) is internationally recognized for developing evidence-based Clinical Practice Guidelines (CPGs) for managing cancer or treatment–related toxicities, but their uptake remains uncertain. This study aimed to evaluate global awareness, utilization, and perceptions of MASCC CPGs, to inform strategies to enhance future updates and promote their implementation.

**Methods:**

An international online survey was distributed via the MASCC platform between February and March 2025 to healthcare professionals specialized in supportive care in cancer. The survey included both closed- and open-ended questions addressing awareness, usage, perceived strengths and limitations, implementation challenges, and suggestions for improvement.

**Results:**

A total of 423 responses were received, 264 (63%) from MASCC members. Participants represented all world regions, with 271 (65%) having 11–20 or more years of clinical experience. Overall, 302 (78%) reported routinely using MASCC CPGs in the management of cancer or treatment-related toxicities. Even among non-members, MASCC CPGs were cited as the most frequently used guidelines compared with other sources. The mucositis and antiemetics CPGs were the most recognized and applied. Key facilitators included the guidelines’ strong evidence and multidisciplinary development. Reported barriers included limited availability or high cost of recommended interventions. Respondents suggested improvements related to the timing of updates, dissemination strategies, and the inclusion of patient-facing guidelines.

**Conclusions:**

MASCC CPGs are widely recognized and valued globally, yet implementation remains variable. Targeted efforts to address resource constraints and enhance dissemination may strengthen their global awareness and implementation.

**Supplementary information:**

The online version contains supplementary material available at https://doi.org/10.1007/s00520-026-11388-1.

## Background

The Multinational Association of Supportive Care in Cancer (MASCC) is a leading international organization dedicated to advancing evidence-based supportive care for individuals affected by cancer and its treatment. A central component of MASCC’s mission is the development of Clinical Practice Guidelines (CPGs) that synthesize the best available evidence to guide the prevention and management of treatment-related toxicities. These evidence-based CPGs, developed by multidisciplinary expert panels, aim to improve patient outcomes [1–3], standardize best care practices, and support clinical decision-making across diverse healthcare settings.

Over the past decades, MASCC has produced several influential CPGs covering major domains of supportive care, including mucositis [4], antiemetics [5], radiation dermatitis [6], osteoradionecrosis (ORN) [7], medication-related osteonecrosis of the jaw (MRONJ) [8], salivary gland hypofunction [9], survivorship care [10–12] and other treatment-related complications [13–17]. These CPGs have shaped standards of care worldwide and in many MASCC Centers of Excellence, institutions recognized for exemplary application of evidence-based supportive care, serving as models of high-quality practice that promote multidisciplinary collaboration, CPGs adoption, and knowledge exchange to enhance patient outcomes [18]. However, the degree to which MASCC CPGs are known, adopted, and implemented within and beyond MASCC Centers of Excellence has not been comprehensively examined. Variation in healthcare infrastructure, resource availability, and awareness may all influence implementation and limit the global impact of these evidence-based recommendations.

In recent years, there has been a growing interest in strategies for effective implementation and dissemination of CPGs [19–23]. Understanding the real-world awareness, accessibility, and perceived utility of MASCC CPGs among clinicians is essential to ensuring their continued relevance and reach. Previous studies on clinical CPGs implementation in oncology and supportive care highlight common barriers, including a lack of dedicated and structured implementation resources, limited dissemination, insufficient contextual adaptation, and cost or feasibility constraints, particularly in resource-constrained settings [19–22, 24, 25]. Identifying such barriers and facilitators from a global perspective can guide targeted strategies for future CPGs development, dissemination, and implementation, thereby improving patient outcomes for all people affected by cancer.

This study, therefore, aimed to assess global awareness, use, and perceptions of MASCC CPGs among healthcare providers involved in supportive cancer care. To design effective strategies for implementing supportive care CPGs, the authors investigated the factors that promote their use and the challenges that affect their implementation. Insights from this survey may inform future CPGs updates and support MASCC’s ongoing efforts to optimize the translation of evidence into clinical practice worldwide.

## Methods

### Survey development

The survey was developed by the Mucositis Study Group (MSG) leadership team (PB, HRW) in consultation with experts from multiple supportive care disciplines who are members of the MASCC MSG Guidelines Advisory Board. Inclusion criteria included professionals who provide care for people with cancer. Exclusion criteria included individuals who did not provide answers to any survey items. These criteria describe participant eligibility and not survey-development procedures. Questions were designed to elicit both quantitative and qualitative insights into CPG awareness, use, perceptions, and implementation challenges. The survey comprised 50 questions, including multiple-choice, Likert-scale, and open-ended items, required approximately 20 min to complete, and was organized into five sections:

1. Demographics and professional background (e.g., country, discipline, years of experience, MASCC membership status)
2. Awareness and utilization of MASCC CPGs
3. Implementation and perceived strengths and limitations of MASCC CPGs
4. Recommendations for improving dissemination and future updates
5. Use and perception of specific MASCC CPGs

In this study, the term “MASCC Guidelines/CPGs” refers to guidelines either developed independently by MASCC, or collaboratively with other organizations, namely the International Society for Oral Oncology (ISOO) [4, 7–9], the American Society of Clinical Oncology (ASCO) [7–9, 11, 12], and European Society for Medical Oncology ESMO [5].

The draft survey was pilot tested with members of the MASCC Guidelines Advisory Board and subsequently revised by the MASCC Executive Committee and Study Group Chairs to ensure clarity, content validity, and appropriate completion time. Feedback-informed refinements to item wording and response options. The final survey is provided in Supplement 1. The study was reported in accordance with the Checklist for Reporting of Survey Studies (CROSS) [26], provided in Supplement 2.

### Survey distribution and participants

The cross-sectional survey was distributed electronically via the MASCC communication platform, including the organization’s email list and social media channels, between February 18 and March 14, 2025. Further dissemination occurred when individuals shared or re-posted the survey link, allowing it to reach non-MASCC members and broader professional audiences. Two weeks after the initial invitation, a reminder email was sent. Participation was voluntary and open to healthcare professionals engaged in supportive care in oncology, including physicians, nurses, dentists, and allied health professionals (e.g., pharmacists, social workers, dietitians, and clinical exercise professionals). Respondents were informed about the study’s purpose, anonymity, and confidentiality prior to participation. No incentives were provided. A convenience sampling approach was utilized, allowing any eligible respondents to participate by accessing the survey link. Therefore, it was not possible to define the total sampling frame or calculate a predetermined sample size. To minimize multiple participation, survey access was restricted to one submission per device.

### Data collection and analysis

Survey data were collected using the SurveyMonkey platform (San Mateo, CA, USA) and exported for analysis. Quantitative data were analyzed descriptively using frequency distributions and proportions. Comparisons by MASCC membership were performed using cross-tabulation. Open-ended responses were analyzed thematically to identify common perceptions, barriers, facilitators, and suggestions for improvement. Participants could opt out of individual questions; unanswered items were treated as missing data and excluded from the corresponding analyses. Differences in CPGs awareness and use between collaborative and single-organization (MASCC) CPGs were compared using Welch's t-test, using R version 4.4.1 (R Foundation for Statistical Computing, Vienna, Austria), and *p* < 0.05 was considered statistically significant.

### Ethical considerations

The study was conducted in accordance with the ethical standards of MASCC and the Declaration of Helsinki. The study was approved by the Institutional Review Board of Rambam Health Care Campus, Haifa, Israel (RMB-0386-24). The survey did not gather personal identifiers or sensitive information. Participation in the survey was voluntary and anonymous; consent was deemed provided. All responses were stored securely and handled confidentially. Participants could withdraw from the survey at any time, and any data entered up to the point of withdrawal was retained.

## Results

### Characteristics of survey respondents

A total of 423 respondents participated in the survey; 268 respondents had full responses to all questions. Institutional affiliation, professional background, and geographic distribution of respondents are summarized in Table 1.

**Table 1:** Institutional affiliation, professional background, and geographic distribution of survey respondents

	Category	Number; Percentage (%)
Institutional Affiliation	University hospitals	129 (31%)
	Tertiary care centers	71 (17%)
	University faculty	62 (15%)
	Governmental hospitals	48 (11%)
	Private practice or hospital	45 (10%)
	Community hospitals	29 (7%)
	Other	38 (9%)
Professional Role	Physicians	178 (42%)
	Dentists/Oral Medicine	110 (26%)
	Nurses	44 (10%)
	Pharmacists	15 (4%)
	Faculty members	14 (3%)
	Physical therapist*	11 (2.6%)
	Dietitian	7 (1.7%)
	Other	40 (9.5%)
Clinical Specialty	Oral medicine/Maxillofacial Surgery/Dentistry	113 (27%)
	Medical oncology	90 (22%)
	Nursing	40 (10%)
	Palliative care	36 (9%)
	Allied health professions	22 (5%)
	Radiation oncology	14 (3%)
	Hematology	13 (3%)
	Other	89 (21%)
Years of Experience	> 20 years	140 (33%)
	11–19 years	131 (31%)
	6–10 years	76 (18%)
	0–5 years	73 (17%)
Patient Population#	Adults	372 (89%)
	Adolescents and young adults (15–21 years)	102 (24%)
	Children (0–14 years)	69 (16.5%)
Primary Care Setting	Outpatient	193 (46%)
	Inpatient	100 (24%)
	Mixed (outpatient, inpatient, home care)	87 (21%)
	Home care	7 (1.7%)
Geographic Region	Western Europe	110 (26%)
	South America	65 (15.4%)
	Pacific/Oceania region	64 (15.2%)
	North America	53 (13%)
	South and Southeast Asia	41 (9.7%)
	East and Central Asia	35 (8.3%)
	West Asia/Middle East	23 (5.5%)
	Eastern Europe	11 (2.6%)
	Africa	8 (1.9%)
	Other	10 (2.4%)

*The term “Physical Therapist” was used in the survey; however, this may encompass Clinical Exercise Professionals, including Physical Therapists, Exercise Physiologists, Occupational Therapists, and Kinesiologists

#Multiple answers possible

### Awareness and utilization of MASCC and other supportive care CPGs

**Fig. 1:**
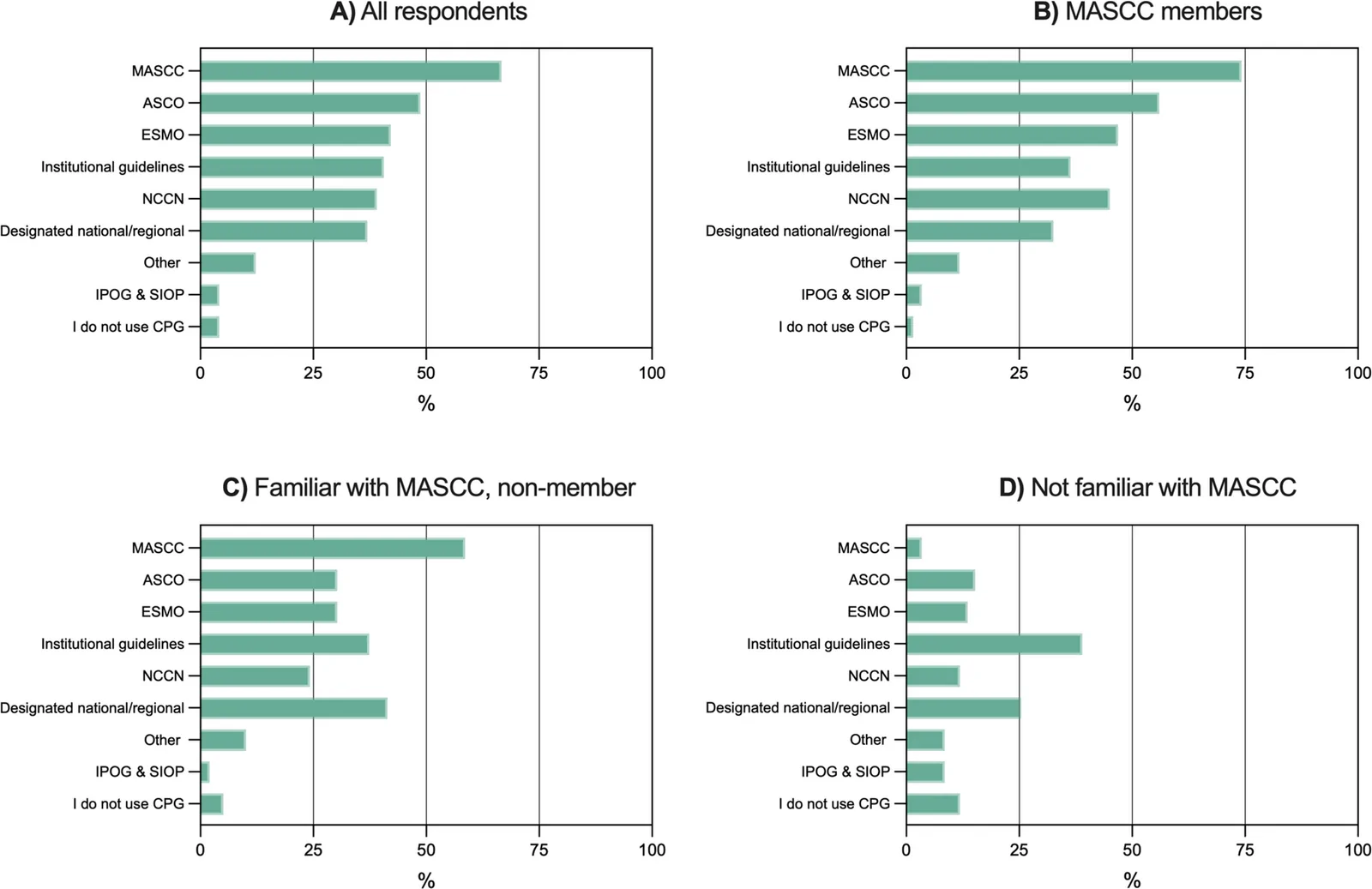
**A** Most frequently used CPGs in clinical practice, by organization, among all respondents. *N* = 422. **B** Most frequently used CPGs, among MASCC members. *N* = 264. **C** Most frequently used CPGs, among non-MASCC members, who are familiar with MASCC. *N* = 99. **D** Most frequently used CPGs, among respondents who are not familiar with MASCC. *N* = 59. Multiple selections possible. ASCO – American Society of Clinical Oncology; CPG – Clinical Practice Guidelines; ESMO – European Society for Medical Oncology; IPOG – International Pediatric Oncology Group; MASCC – Multinational Association of Supportive Care in Cancer; NCCN – National Comprehensive Cancer Network; SIOP – International Society of Paediatric Oncology

Overall, 63% of respondents were MASCC members, 24% were familiar with MASCC but not members, and 14% reported no familiarity. Respondents were asked if they used any CPGs in the management of toxicities related to cancer or its treatment, with 78% reporting routinely applying them, while 15% used them occasionally. The most frequently used CPGs included those issued by MASCC (67%)**,** ASCO (49%)**,** ESMO (42%), institutional guidelines (41%), and National Comprehensive Cancer Network (NCCN, 39%) (Fig. 1). Among respondents who were familiar with MASCC but not members, MASCC CPGs were the most frequently used (59%), followed by national guidelines (41%), institutional guidelines (37%), ASCO guidelines (30%), and ESMO guidelines (30%). In contrast, participants unfamiliar with MASCC primarily relied on institutional (39%) or national guidelines (25%), with lower usage of ASCO (15%), ESMO (14%), or NCCN guidelines (12%), and 11% reporting not using any CPGs. The most frequently used CPGs by organization among respondent groups are presented in Fig. 1. Notably, respondents were asked which organizations’ CPGs they use in clinical practice, without distinguishing between guidelines developed by individual organizations and those developed collaboratively.

**Fig. 2:**
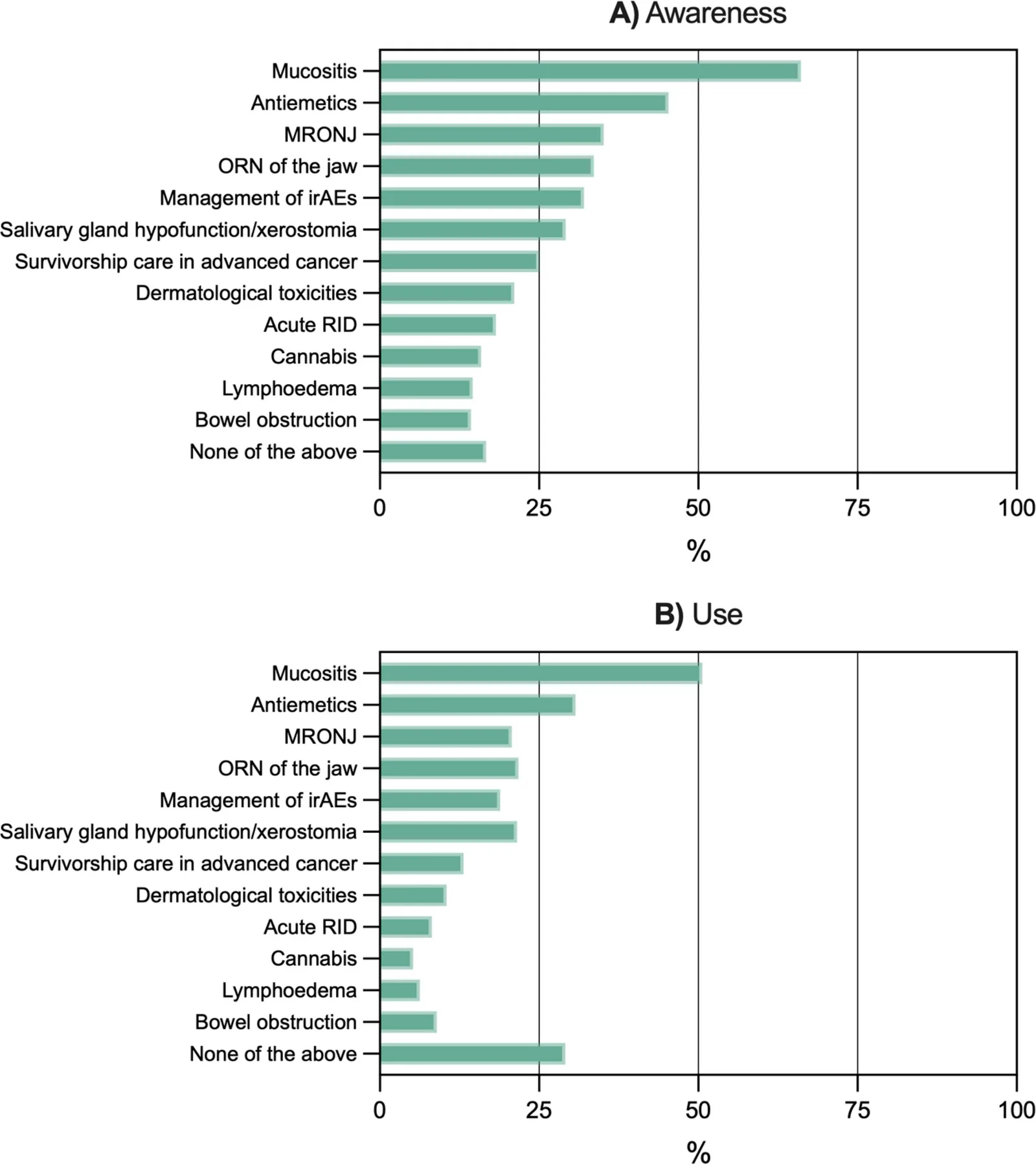
**A** Awareness of MASCC clinical practice guidelines among respondents. Multiple selections possible. **B** Usage of MASCC clinical practice guidelines among respondents. Multiple selections possible. irAE- immune-related adverse events. MRONJ- Medication related osteonecrosis of the jaws. ORN- Osteoradionecrosis. RID- Radiation-induced dermatitis

Awareness of MASCC CPGs varied across domains. Respondents were asked which MASCC CPGs for supportive cancer care they were aware of, with the mucositis CPGs receiving the highest level of recognition (66%)**,** followed by antiemetics (45%)**,** MRONJ (35%)**,** ORN of the jaws (34%), immune-related adverse events (32%), and salivary gland hypofunction (29%) (Fig. 2A).

Likewise, the respondents were asked which MASCC CPGs they routinely use across supportive care areas. The most applied CPGs were for mucositis (51%)**,** antiemetics (31%)**,** ORN of the jaws (22%), salivary gland hypofunction (22%), and MRONJ (21%) (Fig. 2B). Consistent with patterns of awareness, CPGs utilization differed by topic, with higher use for mucositis and antiemetics. Notably, routine use rates for each CPG were generally lower than the corresponding awareness rates.

When comparing awareness and use between collaborative and single-organization MASCC CPGs, collaborative CPGs were associated with significantly higher reported average awareness (39.1% vs. 19.4%, *p* < 0.001), and use (26.4% vs. 9.7%, *p* < 0.001), than single-organization MASCC CPGs (Online Supplementary Fig. 1).

### Implementation, and perceived strengths and limitations of MASCC CPGs

Less than half of respondents (46%) reported that MASCC CPGs are formally incorporated into their institutional or national guidelines and protocols. Among those who use MASCC CPGs, most noted a high degree of similarity with local guidance: 2% described them as “almost identical,” 23% as “very similar,” 28% as “similar,” and 15% as “a little different.”

Respondents generally held positive perceptions of MASCC CPGs, highlighting several key strengths. The majority agreed that MASCC CPGs are developed by experienced multidisciplinary experts (94%), are evidence-based (89%), clinically relevant (87%), comprehensive (79%), and useful in daily practice (79%). Additionally, 74% considered them well-structured and easy to use, 72% noted that they help increase awareness of new therapies, and 69% felt they were unbiased. Despite these strengths, several areas of improvement were identified. About 32% of respondents noted that some of the recommended interventions are unavailable or too expensive, and 21% highlighted a lack of reimbursement for certain CPGs-recommended treatments. Thirty-eight percent agreed that MASCC CPGs are superior to institutional guidelines, 61% found them easy to access, and 68% agreed that updates are timely. Importantly, only 21% believed MASCC CPGs influence insurance/healthcare coverage of additional therapies, and 28% felt they are developed by a limited number of professionals.

### Recommendations for improving dissemination and future updates

Respondents provided a range of suggestions to improve MASCC CPGs development, dissemination, and accessibility. Regarding the timing of updates, 41% preferred a major update every five years supplemented by updates in response to major new evidence (e.g., high-quality randomized controlled trials) and periodic short summaries every one to two years. Meanwhile, 35% favored updates every five years plus updates for major changes, 17% preferred updates only when new evidence emerges, and 6% supported major updates every five years only (Online Supplementary Fig. 2).

Respondents emphasized the need for ongoing communication through various channels, such as apps, newsletters, conferences, and partnerships with patient and cancer advocacy organizations, as well as other CPG bodies like ASCO, ESMO, and NCCN. Specific dissemination preferences included publication in the *Journal of Supportive Care in Cancer* (64%), collaboration with professional organizations (58%), webinars (53%), interactive digital platforms such as mobile apps (47%), and social media (44%) (Online Supplementary Fig. 3).

In terms of CPGs access, 55% preferred online websites, 26% favored using a mobile app, and 13% cited a scientific journals. Participants also emphasized the need for a simplified and visual format, including infographics, flowcharts, summaries, digital formats, and person-centered and friendly materials. Additional expectations included broader accessibility through open access, translations to languages other than English, inclusion of diverse healthcare roles (e.g., nurses, pharmacists, allied health professionals), and regional adaptation to account for resource-limited settings and local clinical realities.

Regarding patient-facing CPGs, most respondents (78%) supported sharing adapted versions with patients to ensure comprehension and accessibility, suggesting formats such as webinars, advocacy group partnerships, social media, patient-friendly summaries, booklets, and digital platforms. A smaller proportion believed CPGs should remain strictly for healthcare providers (17%), while 5% supported sharing CPGs with patients without adaptation.

Respondents provided a range of suggestions for topics to address in future MASCC CPGs. The most frequently cited areas included risk stratification and prediction (65%) and assessing or grading symptoms and treatment-related side effects (56%), followed by financial toxicity (35%), linking preclinical findings to clinical practice (29%), and patient navigation (28%). Additional suggestions encompassed a broad spectrum of supportive care needs, including behavioral interventions and tailored exercise prescriptions, rehabilitation, management of depression, cancer-related cognitive impairment, neurological toxicities, spiritual needs, survivorship care, telehealth and digital care models, palliative and end-of-life care, and considerations for low-resource or community settings and equity in care.

### Use and perception of specific MASCC CPGs

**Fig. 3:**
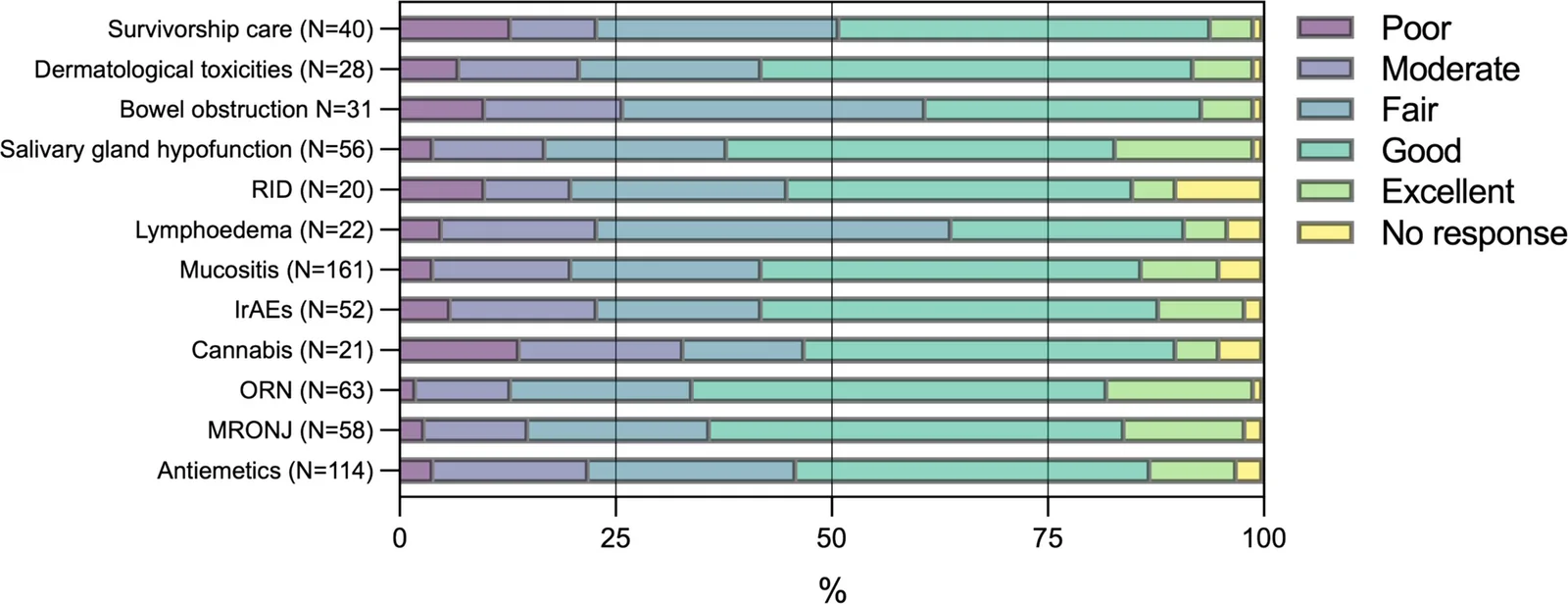
Reported levels of implementation for individual MASCC CPGs. IrAEs- immune-related adverse events. MRONJ- Medication Related Osteonecrosis of the Jaws; ORN- Osteoradionecrosis. RID- Radiation-induced dermatitis

Respondents were asked to select up to three MASCC CPGs they were most familiar with or used most frequently in their daily clinical practice and wished to discuss. Of all CPGs, 56% chose mucositis**,** 40% antiemetics**,** 22% ORN, and 20% MRONJ, reflecting a pattern consistent with overall CPGs usage frequency. Respondents were asked to rate the implementation of the MASCC CPGs they had selected previously. Overall, 45% rated CPGs implementation as excellent or good, 22% as fair, 18% as poor or moderate, and 15% reported that they do not use MASCC CPGs. Implementation ratings for individual MASCC CPGs, based on survey responses are illustrated in Fig. 3. The level of implementation closely mirrors the knowledge and dissemination of each CPG.

**Fig. 4:**
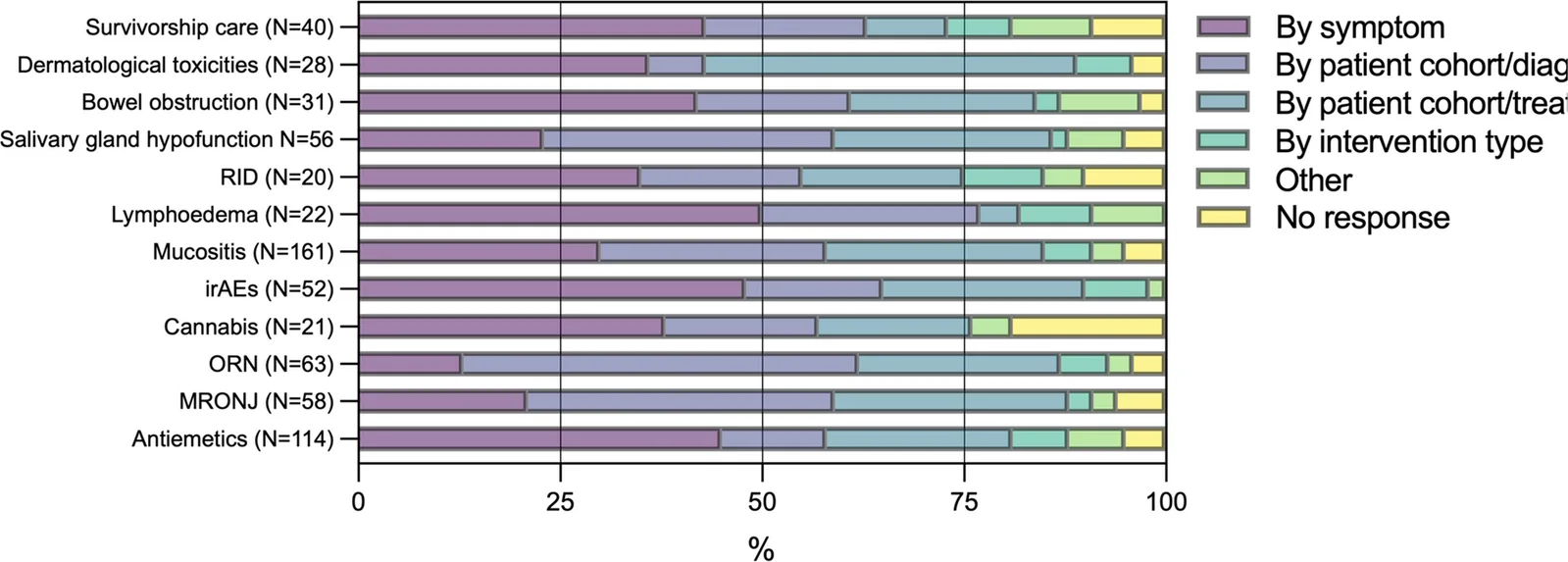
Respondents' preferences on the structure of CPGs for optimal use. IrAEs- immune-related adverse events. MRONJ- Medication Related Osteonecrosis of the Jaws; ORN- Osteoradionecrosis. RID- Radiation-induced dermatitis

When asked about the optimal structure of the CPGs for practical use, respondents most frequently suggested organization by symptom (31%)**,** patient cohort/diagnosis (22%)**,** patient cohort/treatment type (21%)**,** intervention class/type (6%), and other formats (6%), while 13% indicated they do not use MASCC CPGs. Respondents’ recommendations for structuring CPGs to maximize usability are illustrated in Fig. 4.

## Discussion

This international survey provides a comprehensive overview of healthcare professionals’ awareness, use, and perceptions of MASCC Clinical Practice Guidelines (CPGs), offering valuable insights for future CPGs development, dissemination, and implementation strategies. Overall, MASCC CPGs are widely recognized and valued among supportive care providers, including both MASCC members and non-members. Respondents perceive MASCC CPGs as being evidence-based, clinically relevant, comprehensive, and user-friendly, with strong multidisciplinary development, a foundation that supports their credibility and adoption in clinical practice.

Awareness of MASCC CPGs varied across domains. These findings indicate that while some CPGs are widely known, overall awareness differs depending on the specific supportive care area addressed by MASCC. The highest awareness and use were reported for mucositis and antiemetic CPGs, suggesting that CPGs covering common or high-impact toxicities could be somewhat more widely recognized. This may also reflect both the historical leadership and perceived authority of MASCC in these specific topics. The MASCC/ISOO mucositis CPGs, for instance, represent one of the earliest and most high-profile efforts to synthesize evidence on prevention and management strategies, and they remain the primary international reference cited by other societies. Similarly, the MASCC antiemetic CPGs have a long-standing legacy of authority, which is compounded by collaboration with ESMO, with the two societies issuing jointly endorsed recommendations. Our findings indicate that collaborative CPGs were associated with significantly higher reported awareness and use than single-organization MASCC CPGs. Collaboration across professional organizations may enhance visibility, credibility, and dissemination by providing access to multiple professional networks, communication channels, and clinical communities, thereby increasing opportunities for implementation. These findings support continued collaboration between MASCC and other professional organizations as a potentially useful strategy for broadening the reach of supportive care recommendations, reinforcing trust, and facilitating their wider dissemination. However, this association should be interpreted cautiously, as differences in CPGs topics, target audiences, or dissemination strategies may also contribute to the observed differences. Additionally, it is important to note that CPGs awareness may be influenced by their publication age, with older guidelines having more time for circulation, dissemination, and adoption.

In other supportive care areas, multiple overlapping CPGs (e.g., from NCCN, ESMO, or national oncology groups) coexist, potentially diluting awareness of MASCC’s distinct role. MASCC's strength lies in its global, multidisciplinary approach, which encompasses a wide range of healthcare professionals. This distinguishes it from other societies and emphasizes the importance of evidence synthesis over consensus-based recommendations. However, this focus can also limit its visibility in regions or institutions that primarily rely on local or society-specific guidelines. Most respondents reported that their local guidelines were “identical,” “almost identical,” or “very similar” to MASCC's CPGs. The observed similarity between local protocols and MASCC CPGs may imply incorporation of MASCC guidance into institutional practice; however, this cannot be established from our survey. Local protocols may instead reflect recommendations from multiple professional organizations, institutional expertise, or broader evidence-based practice, and different guideline sources may provide concordant recommendations. Future studies linking specific institutional protocols to their cited sources could help clarify the contribution of MASCC CPGs to local implementation. However, formal incorporation of MASCC CPGs into institutional or national protocols remains limited, highlighting an opportunity for MASCC to actively engage with national and society guideline developers to develop joint guidelines aligned to local contexts.

Although respondents identified barriers such as limited availability or high cost of recommended interventions, difficulties accessing CPGs, and lack of reimbursement, these challenges are not unique to MASCC. Similar limitations have been reported across oncology and supportive care CPGs from ASCO, ESMO and the NCCN [23, 27–29], underscoring the broader challenge of translating evidence-based recommendations into routine practice. Importantly, meaningful implementation requires changes in healthcare professionals’ attitudes and behaviors, which are difficult to achieve; however, when adherence to supportive care CPGs can be linked to improvements in clinically relevant outcomes, supportive care becomes a clear contributor to higher-quality cancer care[30]. Implementation initiatives in other settings such as ESMO’s Clinical Benefit Framework, have demonstrated that uptake improves when CPGs are accompanied by implementation tools, educational resources, and mechanisms for local adaptation and adoption [23, 27, 31, 32]. Delivering best-practice cancer care requires structured implementation efforts and adequate resourcing. While clinician training is a common strategy, it is insufficient on its own and is most effective when combined with additional approaches such as local champions, prompts and reminders, organizational support, and embedding recommendations into electronic health records (EHRs) [33–36]. For MASCC, these examples suggest that going beyond the development of CPGs by strengthening implementation pathways could enhance visibility and use. Importantly, addressing cost and reimbursement barriers requires tailoring recommendations to the context of each healthcare system [37].

Consideration of financial toxicity, at the patient and system level, is essential to ensure CPG recommendations are feasible, equitable, and sustainable. The inclusion of cost-effectiveness analyses within CPGs could assist healthcare systems facing financial constraints in prioritizing and justifying the adoption of evidence-based interventions. ASCO and NCCN similarly recognizes socioeconomic and structural barriers, and have developed resource-stratified CPGs to facilitate local adaptation and improve relevance in diverse settings [27]. The recently published MASCC antiemetic consensus recommendations for resource-limited settings provide a timely example of adapting evidence-based guidance to diverse healthcare contexts [38]. Given substantial global variability in healthcare resources, creating a framework for resource stratification should be a priority for MASCC to enhance the global applicability and implementation of CPGs across diverse settings and healthcare systems with varying social, structural, and economic circumstances.

Although awareness of MASCC CPGs is high, their routine use differs across supportive care areas. Notably, routine use rates for each CPG were generally lower than the corresponding awareness rates, highlighting the well-recognized knowledge-to-practice gap in supportive oncology [39–41], which is influenced by several interconnected factors. First, awareness does not necessarily equate to familiarity: clinicians may be aware of a CPG’s existence but lack a detailed understanding of its recommendations or their practical application [31, 42]. Second, attitudes towards the CPGs can influence uptake: clinicians may disagree with specific recommendations, lack confidence in implementing them, or remain unconvinced that CPG adherence leads to meaningful improvements in patient care [42]. Providers may not view certain interventions (e.g., exercise counseling) as part of their professional role, despite agreeing with their benefits, and lack knowledge of referral pathways [43]. Third, various organizational and external barriers are often encountered, including limited time, insufficient staffing or resources, lack of reimbursement, perception of clinician roles, and the absence of supportive systems, such as electronic reminders, to facilitate the implementation of CPGs [31, 42]. Patient-level barriers, such as financial constraints or difficulty accessing supportive services, further impede CPG uptake, demonstrating that knowledge alone does not ensure equitable or consistent application [43, 44]. Finally, the degree to which CPGs align with existing clinical workflows affects their use: lack of integration into routine processes makes recommendations overly complex or time-consuming, hampering regular implementation [45, 46]. Even with financial incentives, compliance with oncology pathways remains suboptimal [46]. Thus, awareness of supportive oncology CPGs is necessary but not sufficient for routine clinical use due to barriers related to knowledge, attitudes, resources, workflow, and patient factors [29, 31, 32, 47].

Putting these findings into perspective over time highlights the substantial progress in CPGs awareness and use over the past two decades. A survey conducted by leaders of the MASCC Mucositis Study Group in 2006 [48], following the 2004 mucositis CPGs, found that only 7% of respondents were using the guidelines and 33% were aware of them. In contrast, the current survey reports 51% use and 66% awareness, demonstrating a marked increase over time. These trends highlight that building a recognized and trusted CPGs “brand” is a cumulative process that strengthens with each update, gradually enhancing awareness and adoption.

Respondents emphasized the importance of CPGs structure and accessibility. Preferred formats included symptom-based organization, patient cohort or diagnosis/oncological treatment stratification, and concise visual aids such as flowcharts and summaries; only a minority reported the preferred format being related to intervention type. Interestingly, only 61% of respondents reported that CPGs are easy to access, despite most MASCC CPGs papers being published as Open Access and several CPGs including translated versions. Digital tools, including interactive platforms and apps, were highlighted as important avenues for dissemination, alongside traditional methods such as journals, webinars, conferences, and collaborations with organizations like ASCO, ESMO, and NCCN. The potential role of social media and digital platforms warrants further exploration, particularly in relation to MASCC’s existing digital reach. Future efforts could focus on structured dissemination strategies, including pocket reference cards, app-based clinical algorithms, and EHR-embedded decision support tools to facilitate uptake. MASCC and ASCO have created artificial intelligence (AI) guideline assistants to navigate people with queries to best-practice guideline-driven solutions [49, 50]. These user-friendly formats may also facilitate the identification of practical actions and automatically scan the EHR to identify deviations from recommended care, providing real-time feedback to clinicians and facilitating more consistent implementation of CPGs.

In addition, future CPG development should actively involve patient representatives and advocates in both the design and dissemination phases. Incorporating patient perspectives into future MASCC CPG updates may enhance adherence, promote more holistic and patient-centered care, address real-world needs and communication preferences, strengthen trust and relevance, and support wider dissemination through patient advocacy networks [51–53].

Effective approaches for enhancing healthcare providers’ adherence to CPGs include multifaceted strategies that incorporate academic detailing, audit and feedback, provider reminders, incentive mechanisms and use of digital tools [31, 45, 47]. The use of decision aids and the active involvement of clinic nurses in patient–provider communication further support CPGs implementation by strengthening patient education and engagement [47]. In this context, the MASCC Center of Excellence in Supportive Care in Cancer initiative represents a practical framework for promoting such implementation at the institutional level. By recognizing and supporting centers that demonstrate excellence in the delivery of CPGs-based supportive care, this program facilitates knowledge exchange and fosters institutional commitment. Despite these advances, consistent implementation of supportive oncology CPGs remains challenging. Future research should focus on designing tailored interventions that address system-level barriers, resource limitations, and local context, while also assessing the long-term impact of digital innovations and multidisciplinary strategies on adherence.

Beyond institutional interventions, there is a growing need for strategic engagement at the regulatory and international guideline level. Leadership from MASCC, potentially through its Study Groups, could facilitate collaboration with regulatory bodies such as the U.S. Food and Drug Administration (FDA), European Medicines Agency (EMA) and Therapeutic Goods Administration (TGA) with international guideline developers to promote standardized incorporation of patient-reported outcomes and toxicity monitoring into clinical frameworks. Such alignment can set the stage for standardization of monitoring which would enrich the evidence base underpinning supportive care CPGs [54].

Strengths of this survey include its large, international sample providing a broad perspective across specialties, professional roles, and geographic regions, among MASCC members and beyond, enhancing the generalizability. Nevertheless, findings should be interpreted in light of the study limitations. Specifically, the high proportion of respondents from tertiary centers and highly experienced clinicians, as well as the involvement of MSG initiative and representation of MSG members, including oral medicine specialists and dentists, which may have influenced the survey results toward the mucositis CPGs. It is uncertain how representative the sample is of community or low resource settings. In addition, there may be response bias toward MASCC members or clinicians already familiar with the CPGs, potentially overestimating awareness and use. Participation was voluntary, and individuals with a particular interest in CPGs may have been more likely to respond. As the survey was disseminated primarily through MASCC-related networks and channels, respondents may have been more familiar with MASCC and its CPGs than the broader healthcare community. Additionally, as a self-reported survey, results may not fully reflect actual implementation behaviors in clinical practice. As the survey was disseminated openly via social media and professional channels, it was not possible to independently verify respondents’ professional roles or confirm that all participants were healthcare providers. Given MASCC’s emphasis on global applicability, it may be informative to compare responses from high-income countries versus low- and middle-income countries to explore potential differences in guideline awareness, implementation, and perceived barriers. Approaches that could improve representativeness in future surveys include broader dissemination through collaborating international and regional professional societies, targeted recruitment of underrepresented specialties and geographic regions, systematic sampling where feasible, and monitoring respondent characteristics during recruitment to identify important gaps in representation. Finally, MASCC CPGs are notable for their availability in multiple languages; however, this survey did not assess awareness or use of CPGs based on the availability of translated versions. Future research could explore whether language accessibility influences CPGs awareness and utilization, given its potential impact on global implementation.

In alignment with the Supportive Care 2030 Movement [55], MASCC continues to lead efforts to ensure that high-quality, accessible, and up-to-date management guidelines for treatment-related toxicities, comorbidities, and complications are available and translated into practice. These guidelines are intended to be person-centered, multidisciplinary, and regularly updated to address the evolving profiles of new cancer therapies. Interestingly, several findings from this study mirror the ambitions of the Supportive Care 2030 Movement, which established a global vision for supportive cancer care emphasizing coordinated, patient-centered, and resource-adaptive approaches, comprehensive guideline development, integrated supportive services, patient education and self-management, and strategies to mitigate financial toxicity.

Based on the findings of this study, suggested strategies to further enhance MASCC CPGs implementation are summarized in Table 2.

**Table 2:** Proposed strategies to enhance the implementation of MASCC clinical practice guidelines based on the study findings

1	Engage with national and regional society guideline developers to support harmonized, broader adoption of CPGs, or integration of MASCC recommendations into local settings
2	Develop, in partnership with end-users, practical tools and strategies to ease implementation, including clinician education, locally adaptable resources, and integration into electronic health systems
3	Adopt resource-stratified and graded recommendation models, and explicitly address cost, reimbursement, and financial toxicity, both at the patient and system level, to enhance global feasibility and equity
4	Structure CPGs in multi-modal, concise, user-friendly formats such as symptom-based algorithms (varies by specific CPGs), visual summaries, digital tools (apps, pocket cards, EHR-embedded decision support tools) and social media to facilitate uptake in routine practice
5	Involve patient representatives in CPGs development and dissemination, to enhance trust, relevance and adoption, whilst identifying CPG recommendations that could be directly communicated with patients
6	Develop patient-facing CPGs materials using simplified summaries, webinars, and digital resources to enhance understanding and support shared decision-making
7	Expand MASCC CPGs to address high-priority topics identified by clinicians, including risk stratification and prediction, symptom assessment, financial toxicity, patient navigation, and emerging supportive care needs
8	Continue to promote collaborative CPGs development with international professional organizations as a strategy to broaden the reach, visibility, and dissemination of supportive care recommendations

*CPGs* clinical practice guidelines; *EHR* Electronic Health Records; *MASCC* Multinational Association of Supportive Care in Cancer

In conclusion, MASCC CPGs are widely recognized and valued by healthcare providers worldwide, perceived as evidence-based, clinically relevant, comprehensive, and developed through strong multidisciplinary collaboration. However, their implementation remains variable. Efforts to enhance accessibility, digital dissemination, structured formatting, patient engagement, and adaptation to local contexts are likely to improve CPGs uptake and enhance high quality, evidence-based supportive care. These findings provide actionable insights to guide future MASCC CPGs updates and dissemination strategies, ensuring they meet the evolving needs of both clinicians and people affected by cancer.

## Supplementary information

Below is the link to the electronic supplementary material.

Supplementary file1 (PDF 269 KB)

Supplementary file2 (PDF 445 KB)

Supplementary file3 (PDF 569 KB)

## Data Availability

The datasets used and/or analyzed during the current study are available from the corresponding author on reasonable request.
