# Supplementary material for: Global perspectives on MASCC clinical practice guidelines: awareness, implementation and insights to inform future updates
Source: Support Care Cancer. 2026 Nov 4;34(11):1187. doi: 10.1007/s00520-026-11388-1 (PMC13736418; doi:10.1007/s00520-026-11388-1)
Supplement: Supplementary file 1 [file 520_2026_11388_MOESM1_ESM.pdf]

SURVEY FOR MASCC CLINICAL PRACTICE GUIDELINES

Preface

The Multinational Association for Supportive Care in Cancer (MASCC) is dedicated to ensuring the highest quality care is provided to people with cancer. In line with this goal, MASCC produces a variety of Clinical Practice Guidelines, which aim to provide clear guidance on how to prevent and manage the side effects of cancer and its treatment.

MASCC are committed to ensuring these Guidelines are designed, produced and disseminated most effectively. However, the quality assessment after Guidelines are released is an important step to increase the knowledge about their penetration in the context of real-life clinical practice. This survey aims to identify how people caring for people with cancer use and perceive our Guidelines, and probe various aspects related to their clinical implementation.

This study has been approved by the institutional review board in Rambam Health Care Campus (RMB-0386-24).

The survey can be completed by anyone that provides care for people with cancer. It should take no longer than 20 min. We encourage you to be open and honest, as this feedback will inform how future Guidelines are developed and disseminated.

Thank you for your time.

Kind regards,

Prof Paolo Bossi and A/Prof Hannah Wardill, on behalf of the MASCC Study Group Chair Committee, and the broader MASCC Executive.

The following questions aim to understand more about you and your work environment:

1. Which type of institution(s) do you work in (please select all that apply)?
    - Community hospital
    - Tertiary care cancer center
    - University hospital
    - Private Hospital
    - Private Practice
    - University faculty/teaching
    - Government Hospital
    - Other: ________________________

2. Please select the option that **best** describes your professional role.
    - Physician/medical doctor
    - Physician assistant
    - Advanced practice nurse (e.g. nurse practitioner)
    - Registered nurse/nurse
    - Pharmacist
    - Dietitian/registered dietitian/nutritionist (non-physician)
    - Occupational therapist
    - Physical therapist
    - Social worker
    - Psychologist
    - Dentist/oral medicine
    - Hygienist
    - Faculty
    - Other: ________________________

3. Which is your specialization?
    - Medical oncologist
    - Hematologist
    - Radiation oncologist
    - Palliative care physician
    - Oral medicine specialist or dentist
    - Oral and maxillofacial surgeon
    - Neurologist
    - Pharmacist

- Nurse
- Allied health professional
- Primary care
- Pediatrician
- Dental hygienist
- Other: ________________________

4. How long have you been working in this role?
    - 0-5 years
    - 6-10 years
    - 11-15 years
    - 16-20 years
    - >20 years

5. With which patient population do you primarily work (please select all that apply)?
    - Children (0-14)
    - Adolescents and young adults (15-21)
    - Adults
    - Older adults (aged 65 and above)

6. In what geographic region do you predominantly work?
    - Western Europe (e.g., Greece, Sweden, United Kingdom)
    - Eastern Europe (e.g., Hungary, Poland, Russia)
    - North Africa (e.g., Egypt, Morocco, Sudan)
    - Sub-Saharan Africa (e.g., Kenya, Nigeria, South Africa)
    - West Asia / Middle East (e.g., Iran, Israel, Saudi Arabia)
    - South and Southeast Asia (e.g., India, Indonesia, Singapore)
    - East and Central Asia (e.g., China, Japan, Uzbekistan)
    - Pacific / Oceania (e.g., Australia, Fiji, Papua New Guinea)
    - North America (Canada, United States)
    - Central America and Caribbean (e.g., Jamaica, Mexico, Panama)
    - South America (e.g., Brazil, Chile, Colombia)
    - Other: ________________________

7. In what type of setting do you **predominantly** provide care?
    - Inpatient
    - Outpatient

- Home care
- All of the above equally
- Other: ______________________

8. Are you familiar with the Multinational Association for Supportive Care in Cancer (MASCC)?
    - Yes, I am a MASCC member
    - Yes, but I am not a member
    - No

The following questions aim to understand how you provide care to people experiencing side effects of cancer treatment:

1. Do you use Clinical Practice Guidelines (or similar) to inform how you care for people with cancer experiencing treatment side effects?
    - Yes
    - No
    - Sometimes

2. If you do use Clinical Practice Guidelines, which do you use in the setting of supportive cancer care (please select all that apply)?
    - ASCO
    - ESMO
    - MASCC
    - NCCN
    - IPOG and SIOP for pediatric oncology
    - Designated national/regional guidelines
    - Institutional guidelines
    - Other: _________________
    - I do not use Clinical Practice Guidelines of any kind

3. The Multinational Association for Supportive Care in Cancer (MASCC) produces a range of Clinical Practice Guidelines for various treatment side effects and aspects of supportive cancer care.

    Which **of the following MASCC Guidelines** are you **AWARE** of (please select all that apply)?
    - Antiemetic
    - Medication related osteonecrosis of the jaws

- Osteoradionecrosis of the jaws
- Cannabis for cancer-related pain and/or psychological symptoms
- Management of immune-related adverse events
- Mucositis
- Lymphoedema
- Acute radiation dermatitis
- Salivary gland hypofunction/xerostomia
- Bowel obstruction
- Dermatological toxicities

Survivorship care in advanced cancer

- None of the above

4. Which **MASCC Guidelines** do you currently **USE** in your routine clinical practice (please select all that apply)?
    - Antiemetic
    - Medication related osteonecrosis of the jaws
    - Osteoradionecrosis of the jaws
    - Cannabis for cancer-related pain and/or psychological symptoms
    - Management of immune-related adverse events
    - Mucositis
    - Lymphoedema
    - Acute radiation dermatitis
    - Salivary gland hypofunction/xerostomia
    - Bowel obstruction
    - Dermatological toxicities

    Survivorship care in advanced cancer

    - None of the above

The next set of questions relates to the strengths and limitations of **MASCC's Clinical Practice Guidelines.** Please rate how much you agree or disagree with the following statement.

I value that MASCC Clinical Practice Guidelines are developed by experienced, multidisciplinary professionals.

Strongly agree	Agree	Neutral	Disagree	Strongly disagree

A strength of the MASCC Clinical Practice Guidelines is that they are based on accurate and up to date information, ensuring they are evidence-based.

Strongly agree	Agree	Neutral	Disagree	Strongly disagree

MASCC Clinical Practice Guidelines are easy to find/access.

Strongly agree	Agree	Neutral	Disagree	Strongly disagree

MASCC Clinical Practice Guidelines are produced and updated in a timely manner.

Strongly agree	Agree	Neutral	Disagree	Strongly disagree

MASCC Clinical Practice Guidelines are comprehensive.

Strongly agree	Agree	Neutral	Disagree	Strongly disagree

MASCC Clinical Practice Guidelines are clinically-relevant.

Strongly agree	Agree	Neutral	Disagree	Strongly disagree

MASCC Clinical Practice Guidelines are well structured and easy to use.

Strongly agree	Agree	Neutral	Disagree	Strongly disagree

MASCC Clinical Practice Guidelines increase awareness of new therapies and technologies.

Strongly agree	Agree	Neutral	Disagree	Strongly disagree

MASCC Clinical Practice Guidelines prompt insurance companies and payers to cover additional therapies.

Strongly agree	Agree	Neutral	Disagree	Strongly disagree

MASCC Clinical Practice Guidelines are developed by a limited number of professionals.

Strongly agree	Agree	Neutral	Disagree	Strongly disagree

MASCC Clinical Practice Guidelines are biased.

Strongly agree	Agree	Neutral	Disagree	Strongly disagree

MASCC Clinical Practice Guidelines lack sufficient recommendations for how to PREVENT side effects.

Strongly agree	Agree	Neutral	Disagree	Strongly disagree

MASCC Clinical Practice Guidelines are too heavily focused on what NOT to do.

Strongly agree	Agree	Neutral	Disagree	Strongly disagree

MASCC Clinical Practice Guidelines are NOT useful in daily clinical practice.

Strongly agree	Agree	Neutral	Disagree	Strongly disagree

MASCC Clinical Practice Guidelines are difficult to navigate.

Strongly agree	Agree	Neutral	Disagree	Strongly disagree

Some interventions in the MASCC Clinical Practice Guidelines are not available or too expensive in my country or clinical context.

Strongly agree	Agree	Neutral	Disagree	Strongly disagree

Some interventions in the MASCC Clinical Practice Guidelines require specialist training that is not feasible to access in my country or clinical context.

Strongly agree	Agree	Neutral	Disagree	Strongly disagree

There are legal or national restrictions in my country that prevent me from using MASCC Clinical Practice Guidelines.

Strongly agree	Agree	Neutral	Disagree	Strongly disagree

MASCC Clinical Practice Guidelines methodology is too strict, limiting the ability to provide practical advice in light of limited evidence.

Strongly agree	Agree	Neutral	Disagree	Strongly disagree

MASCC Clinical Practice Guidelines are superior to other Guidelines available at my hospital/center.

Strongly agree	Agree	Neutral	Disagree	Strongly disagree

There is a lack of reimbursement options for the recommendations provided in MASCC Clinical Practice Guidelines.

Strongly agree	Agree	Neutral	Disagree	Strongly disagree

5. Please elaborate on any of the statements above, and your response, if you would like to.

[free text]

6. With respect to the timing of Guideline publication/dissemination, would you prefer:
    - Only a major update according to the criteria used by the existing Guidelines (every 5 years)
    - Major update every 5 years and an update in case of major changes (e.g. landmark RCT published)
    - Major update every 5 years, an update in case of major changes and a periodic (1-2 year short summary about most promising interventions even if not supported by strong evidence)
    - Updates <u>only</u> when new evidence is available
    - Other; ________________________________

7. If you use national, institutional or other international Guidelines/protocols instead of MASCC-specific Guidelines, are MASCC recommendations embedded into these recommendations?
    - Yes
    - No
    - Unsure

8. If you use national or institutional Guidelines/protocols instead of MASCC-specific Guidelines, how similar (or dissimilar) are they to MASCC Guidelines?

- Almost identical
- Very similar
- Similar
- A little different
- Very different
- Completely different
- Unsure
- I do not use national or institutional Guidelines

9. How would you suggest improving the usefulness of the MASCC Clinical Practice Guidelines (e.g. a broader target audience, better dissemination, more frequent updates, or different structure)?

   [free text]

10. Do you believe MASCC Clinical Practice Guidelines should be disseminated with patients/people affected by cancer?
    - No - these guidelines should be strict for healthcare providers
    - Yes, but adaptations should be made to ensure they are accessible and meaningful for patients
    - Yes, they should be shared without adaptations

11. If you believe Guidelines should be disseminated with patients, please explain how you think this could be best achieved (e.g. webinars, cooperation with advocacy groups). [free text].

12. How do you suggest we communicate/disseminate MASCC Guidelines to ensure broad usage (please select all that apply)?
    - Publish in the Journal of Supportive Care in Cancer
    - Publish in a higher impact journal
    - Publish in a more general oncology journal to reach end users beyond outside the supportive care community
    - Webinars
    - Integrate into electronic health systems
    - Integrate with existing platforms that dictate (local) standards of care
    - Build and promote in collaboration with national/international societies/organizations such as ASCO, ESMO, NCCN
    - Interactive digital platform (e.g. an app)
    - Social media platforms
    - Other: ________________

13. What you be the best means to access the guideline in your opinion?

    Scientific journal

    Online website

    Cell phone application

    Recording forms

    Other: __________

14. Do you have any other feedback about MASCC Clinical Practice Guidelines to help us improve our approach to the next update? (e.g. more interactive resources, other recommendations for facilitating clinical use).

    [free text]

The final set of questions aim to understand how you use and perceive a single MASCC Guideline. We kindly ask that you please select the MASCC Guideline that you are most familiar with, or use most in your daily clinical life, and answer the questions with that Guideline in mind.

1. Please select the MASCC Guideline that you would like to discuss.
   - Antiemetic
   - Medication related osteonecrosis of the jaws
   - Osteoradionecrosis of the jaws
   - Cannabis for cancer-related pain and/or psychological symptoms
   - Management of immune-related adverse events
   - Mucositis
   - Lymphoedema
   - Acute radiation dermatitis
   - Salivary gland hypofunction/xerostomia
   - Bowel obstruction
   - Dermatological toxicities
   - Survivorship care in advanced cancer
   - I do not use MASCC Guidelines

2. How would you rate the implementation of the MASCC Guideline (you selected above) in your clinical context?
   - Poor – they are almost never used
   - Moderate – they are used sometimes, but sporadically
   - Fair – they are used but with some adaptations
   - Good – they are used almost all the time
   - Excellent – they are used 100% of the time
   - I do not use MASCC Guidelines

2. With respect to the previous question, please explain the implementation (or lack thereof) of the MASCC Guideline, i.e. if you rated implementation to be poor, what is the reason for this?
   [free text]

3. How would the Guideline be best structured for optimal use?
   - By patient cohort defined by diagnosis (e.g. people with head and neck cancer)
   - By patient cohort defined by treatment type (e.g. people receiving radiotherapy)
   - By intervention class/type (e.g. anti-microbials)
   - By symptom related to the adverse effect (e.g. pain)
   - Other: ______________________

- I do not use MASCC Guidelines

4. What settings and/or situations do you think would benefit from new Guideline(s) being developed (in addition to standard Guidelines that focus on the prevention and management of specific symptoms)? Please select all that apply.
    - Risk stratification/prediction (i.e. how to determine an individual's risk of developing side effects)
    - Financial toxicity (i.e. how to prevent or manage financial implications of cancer and its treatment)
    - Linking preclinical advances to clinical practice
    - How to assess/grade the symptom/side effect of interest
    - Patient navigation
    - Other: _________________

5. How would you suggest improving the usefulness of the specific MASCC Clinical Practice Guideline you selected (e.g. a broader target audience, better dissemination, more frequent updates, different structure)?

    [free text]

6. Do you have any other feedback about the specific MASCC Clinical Practice Guideline you selected to help us improve our approach to the next update?

    [free text]
