# Supplementary material for: Global perspectives on MASCC clinical practice guidelines: awareness, implementation and insights to inform future updates
Source: Support Care Cancer. 2026 Nov 4;34(11):1187. doi: 10.1007/s00520-026-11388-1 (PMC13736418; doi:10.1007/s00520-026-11388-1)

Journal of Supportive Care in Cancer

Global Perspectives on MASCC Clinical Practice Guidelines: Awareness, Implementation and Insights to Inform Future Updates

Ragda Abdalla-Aslan, Paola Alberti, Nicole Blijlevens, Pierluigi Bonomo, Joanne Bowen, Julia S. Bruno, Raymond J. Chan, Yin Ting Cheung, Andrew Davies, Mellar Davis, Sharon Elad, Joel Epstein, Darren Fernandes, Jason L. Freedman, Nicolas H. Hart, Hirotoshi Iihara, Michael Jefford, Daniel Peter Johnstone, Amber S. Kleckner, Bogda Koczwara, Grigorios Kotronoulas, Emma Munro, Barbara Murphy, Midori Nakagaki, Enrique Soto-Perez-de-Celis, Stephen Sonis, Nikolaos Tsoukalas, Anna Ugalde, Corina van der Hurk, Hannah Rose Wardill*, Paolo Bossi*

Corresponding author: Ragda Abdalla-Aslan, Technion Israel Institute of Technology, Technion City, Haifa 3200003. Tel: +972-47772368, Fax: +972-47772557. Email: ragdaa@gmail.com. ORCID: 0000-0002-0649-1512.

Online supplementary figure 1: Comparison of reported awareness and use of collaborative versus single-organization MASCC CPGs. Collaborative CPGs showed significantly higher average awareness (39.1% vs. 19.4%, $p < 0.001$) and use (26.4% vs. 9.7%, $p < 0.001$) than single-organization MASCC CPGs (Welch's t-test).

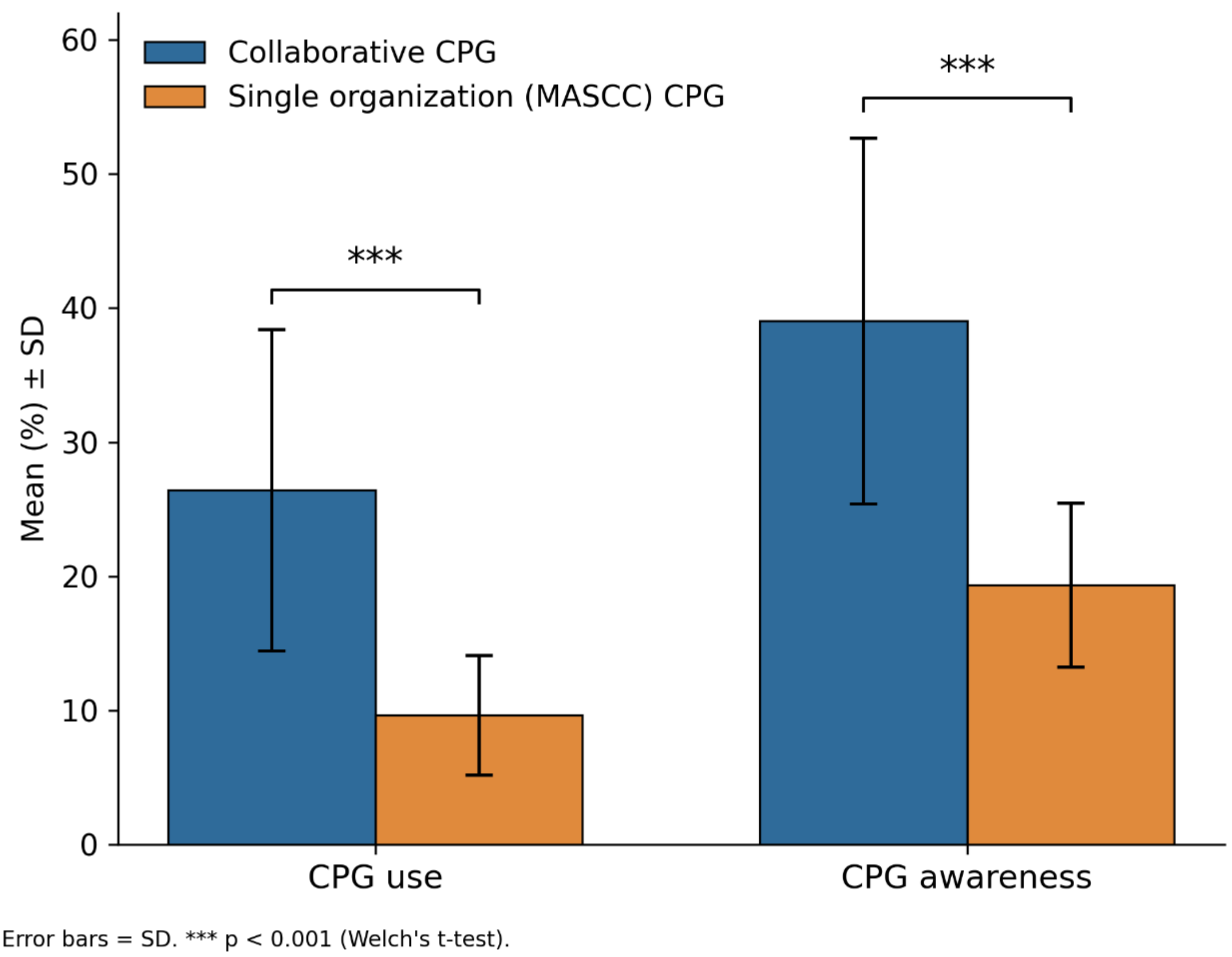

Online supplementary figure 2: Preferences regarding the timing of clinical practice guidelines publication/dissemination.

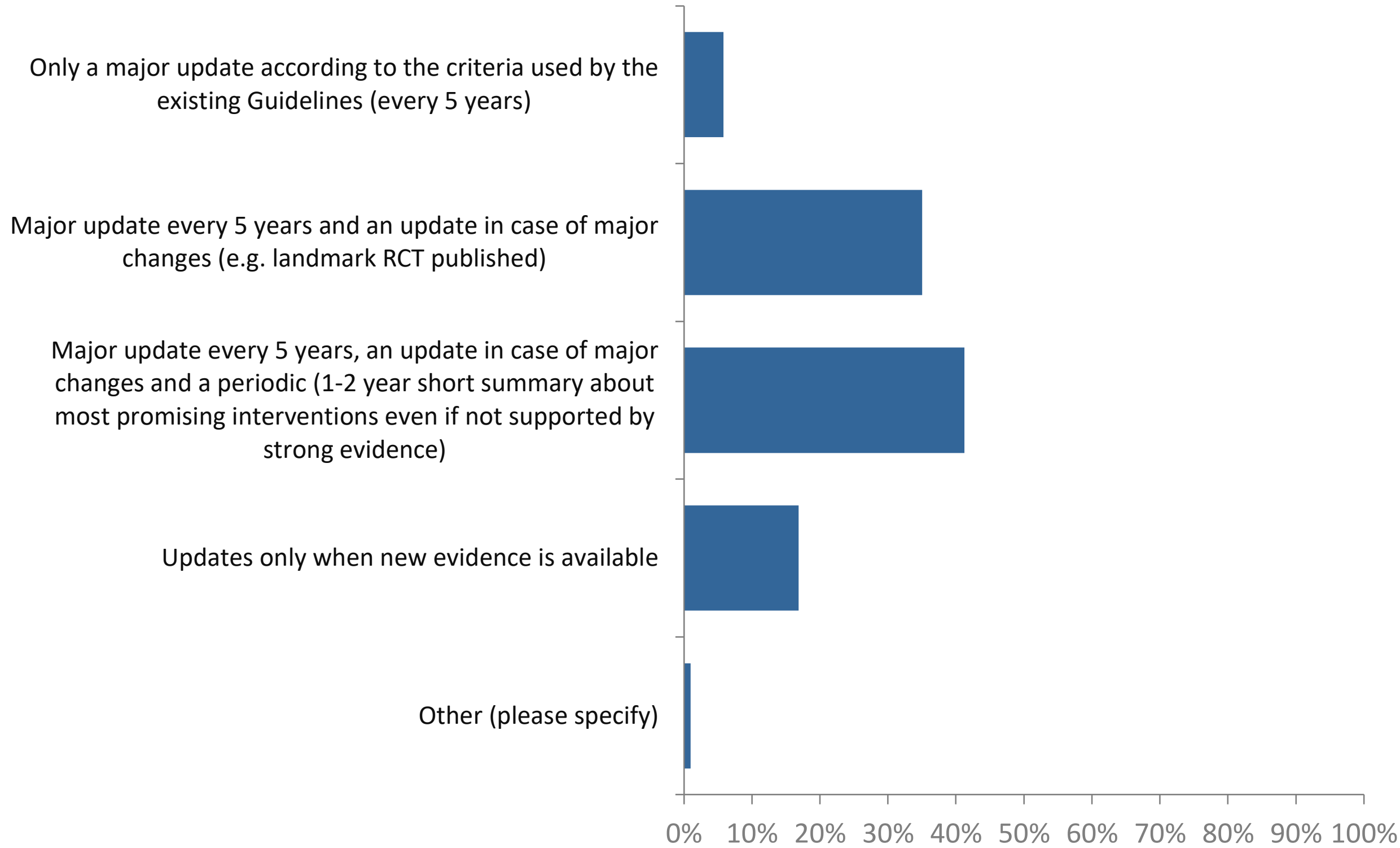

Online supplementary figure 3: Suggestions for dissemination of MASCC CPGs to ensure broad usage. Multiple selections possible.

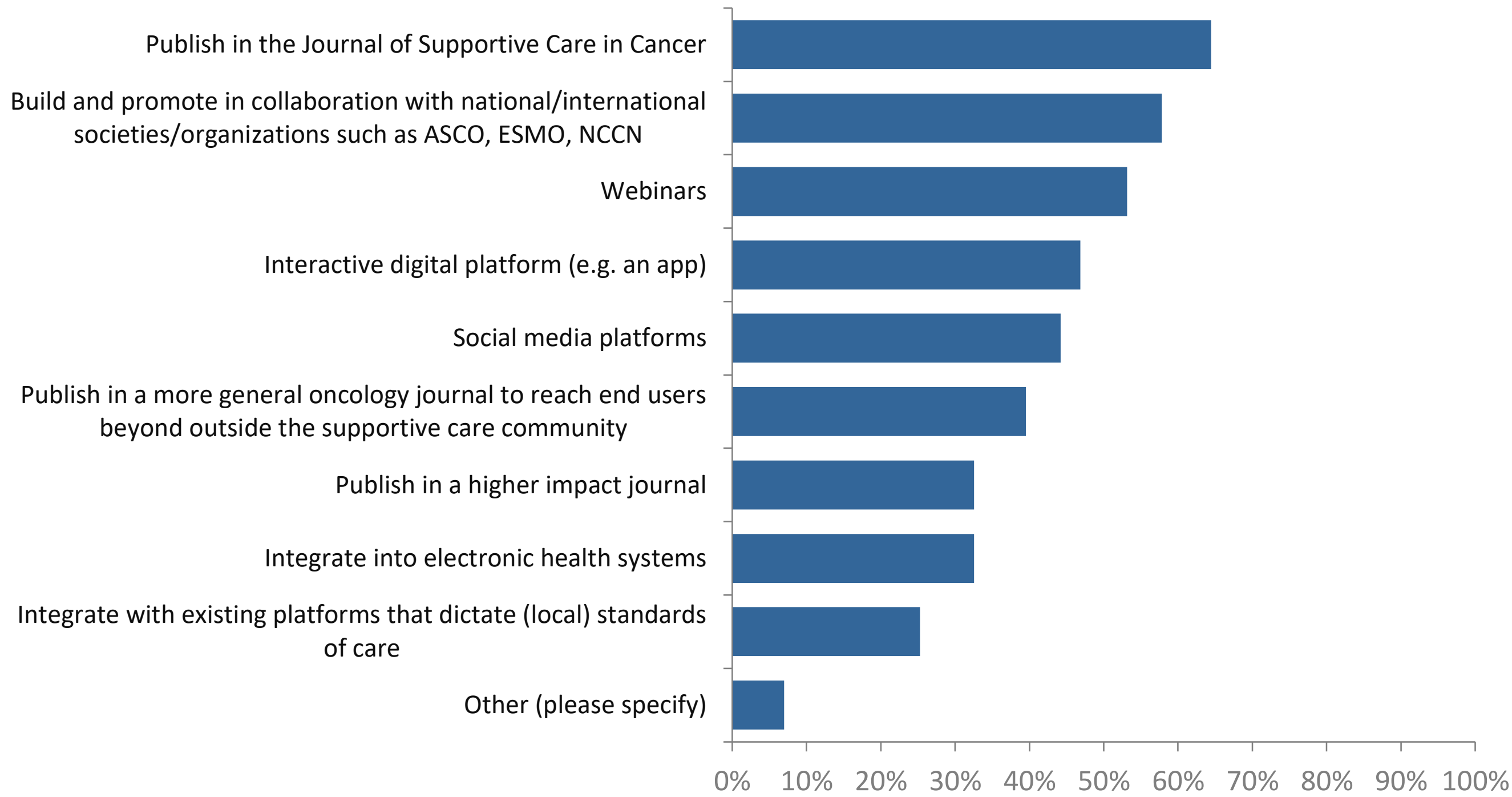

Supplement: Supplementary file 3 [file 520_2026_11388_MOESM3_ESM.pdf]
